# A Density Matrix Renormalization Group Study of the Low‐Lying Excited States of a Molybdenum Carbonyl‐Nitrosyl Complex

**DOI:** 10.1002/cphc.202100549

**Published:** 2021-10-12

**Authors:** Leon Freitag, Leopold Lindenbauer, Markus Oppel, Leticia González

**Affiliations:** ^1^ Institute of Theoretical Chemistry Faculty of Chemistry University of Vienna Währinger Str. 17 1090 Vienna Austria; ^2^ Vienna Research Platform on Accelerating Photoreaction Discovery University of Vienna Währinger Str. 17 1090 Vienna Austria

**Keywords:** density matrix renormalization group, molybdenum, photodissociation, time-dependent density functional calculations, transition metal complexes

## Abstract

A density matrix renormalization group‐self consistent field (DMRG‐SCF) study has been carried out to calculate the low‐lying excited states of CpMo(CO)_2_NO, a molybdenum complex containing NO and CO ligands. In order to automatically select an appropriate active space, a novel procedure employing the maximum single‐orbital entropy for several states has been introduced and shown to be efficient and easy‐to‐implement when several electronic states are simultaneously considered. The analysis of the resulting natural transition orbitals and charge‐transfer numbers shows that the lowest five excited electronic states are excitation into metal‐NO antibonding orbitals, which offer the possibility for nitric oxide (NO) photorelease after excitation with visible light. Higher excited states are metal‐centered excitations with contributions of metal‐CO antibonding orbitals, which may serve as a gateway for carbon monoxide (CO) delivery. Time‐dependent density functional theory calculations done for comparison, show that the state characters agree remarkably well with those from DMRG‐SCF, while excitation energies are 0.4–1.0 eV red‐shifted with respect to the DMRG‐SCF ones.

## Graphical abstract

**Picking orbitals**. An automatized procedure based on the maximum single‐orbital entropy is introduced to select active spaces suitable to describe several electronic excited states simultaneously. The procedure is employed to calculate the low‐lying excited states of CpMo(CO)_2_NO, a molybdenum complex containing NO and CO ligands, using density matrix renormalization group‐self consistent field (DMRG‐SCF) theory.

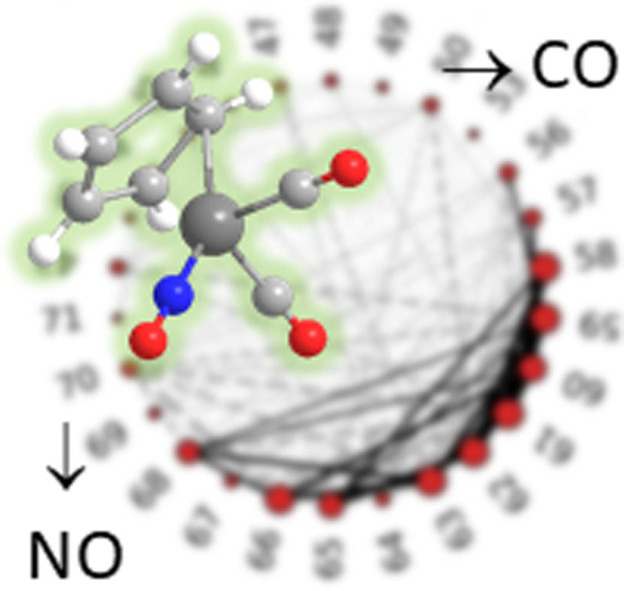

## 1 Introduction

Nitric oxide (NO) is a small molecule known to play important roles in human physiology, such as vasodilation and neurotransmission.[^1^, ^2^, ^3^, ^4^, ^5^, ^6^] It is also a signalling molecule^[7]^ and has shown anticancer activity.[^8^, ^9^, ^10^, ^11^, ^12^] The effect of NO is extremely dependent on its concentration and the tissue in question,[^5^, ^9^] thus, its delivery and release in the tissue in a controlled and targeted manner is a grand challenge. One strategy for controlled NO delivery is the use of NO photoreleasing compounds (photo‐activated NO‐releasing moieties, *photoNORMs*), e. g. in coordinated nitrosyl complexes.[^13^, ^14^, ^15^, ^16^, ^17^, ^18^] In a similar manner, carbon monoxide (CO), although mostly known for its strong toxicity due to strong binding to hemoglobine, was recently discovered to be produced endogenously in small amounts and responsible, similarly to NO, for a variety of physiological effects.^[19]^ This has also triggered the development of targeted CO photoreleasing compounds (*photoCORMs*).[^20^, ^21^, ^22^, ^23^, ^24^, ^25^, ^26^, ^27^]

In order to provide insight into the photodissociation mechanisms of photoNORMs and ‐CORMs and aid the rational design of the precursors, computational studies are useful. In particular, density functional theory (DFT) and its time‐dependent version (TD‐DFT)^[28]^ are the most widespread methods to deal with transition metal complexes^[29]^ and its electronic excited states^[30]^ due to its favourable scaling with system size and thus cheap computational cost. Not surprisingly, they have been also employed to study photoNORMs[^17^, ^31^, ^32^] and ‐CORMs.[^20^, ^23^] However, and despite their appeal and considerable success, DFT and TD‐DFT can perform poorly or even problematic for many transition metal complexes, especially those with significant amount of static correlation.^[33]^ Static correlation is often attributed to the correct description of degenerate (or near degenerate) electronic states, dissociation, or non‐traditional bonding situations. Due to their partially filled *d* sub‐shells, transition metal complexes are predisposed to display many low‐lying nearly‐degenerate states. Kohn‐Sham DFT, by far the most used variant of DFT, is based on a single Slater determinant. Thus, it is unable to properly describe compounds with strong electronic correlation.^[34]^ In such cases, methods that include more than one reference electronic configuration, called *multiconfigurational* or *multireference* methods, are better suited.

Nitric oxide is a *non‐innocent ligand*[^35^, ^36^] and may coordinate to the metal in a linear or bent fashion. Transition metal nitrosyls contain a large amount of static correlation^[37]^ and it is difficult to unambiguously determine an exact oxidation state for the metal and the NO ligand.[^37^, ^38^, ^39^, ^40^, ^41^, ^42^, ^43^, ^44^, ^45^, ^46^, ^47^] Therefore, multiconfigurational methods are well suited and have been successfully employed to study the electronic structure of metal nitrosyl complexes.[^37^, ^38^, ^40^, ^48^] Although CO, unlike NO, usually does not exhibit the behaviour of a non‐innocent ligand, multiconfigurational methods are also well suitable for the studies of excited states and photoreactions of metal carbonyl complexes, including photodissociation.[^49^, ^50^, ^51^, ^52^, ^53^, ^54^]

Among multiconfigurational methods, complete active space self‐consistent field (CASSCF)[^55^, ^56^, ^57^] is probably the most popular one. The CASSCF *ansatz* relies on constructing Slater determinants from all possible electron excitations within a given subset of molecular orbitals, called the *active orbital space*. While conceptually simple, its computational cost scales factorially with the number of active electrons and orbitals: currently, a reasonable limit is about 18 electrons in 18 active orbitals, although it is possible to perform a 20 elecrons in 20 orbitals calculation with a massively parallel setup.^[58]^ One challenge in CASSCF is to chose the active orbitals. As the full valence active orbital space is often too large, the active space must be compromised to be small enough to be feasible but to contain all the necessary orbitals to describe the process at hand. Several rules and guidelines for the active space selection exist,[^59^, ^60^, ^61^, ^62^] but they require *a priori* chemical knowledge on the system, user experience and trial and error, what makes calculations prone to bias and difficult to automatize.

Density matrix renormalization group (DMRG)[^63^, ^64^] for quantum chemistry[^65^, ^66^, ^67^, ^68^, ^69^] provides a remedy to both the computational scaling and the CASSCF active space selection. It allows approximating a CASSCF calculation to an arbitrary accuracy with polynomial scaling with the active space size. Further, orbital entanglement measures[^70^, ^71^, ^72^] from a partially‐converged large‐active‐space DMRG wavefunction, e. g. from a full‐valence space, may be used to select active orbitals in an automated manner.[^73^, ^74^, ^75^, ^76^]

In this work we employ DMRG‐SCF to calculate the absorption spectrum and thus characterise the lowest singlet excited states of CpMo(CO)_2_NO, a transition metal complex with a potential capability to release CO and/or NO upon light irradiation, thus acting as a photoCORM and photoNORM simultaneously.^[18]^ The study of CpMo(CO)_2_NO allows us identifying similarities and differences of CO and NO photodissociation mechanisms. Note that, as showed in other complexes, NO has also the potential to isomerize.[^77^, ^78^, ^79^, ^80^]

### Methods

#### Theory

The DMRG approach^[81]^ iteratively optimizes a wave function ansatz known as the *matrix product state* (MPS)[^82^, ^83^]

(1) $$\left|\left.\Phi \right\rangle \right.=\sum_{\sigma }\sum_{a_{1}=1}^{m}\cdots \sum_{a_{L-1}=1}^{m}M_{1,\alpha_{1}}^{\sigma_{1}}M_{\alpha_{1},\alpha_{2}}^{\sigma_{2}}\cdots M_{\alpha_{L-1},1}^{\sigma_{L}}\left|\sigma_{1}\sigma_{2}\cdots \left.\sigma_{L}\right\rangle \right.$$

where *L* is the total number of orbitals, the occupation number vector $\left|\sigma_{1}\sigma_{2}\cdots \left.\sigma_{L}\right\rangle \right.$ contains the occupation number $\left|\left.\sigma_{l}\right\rangle \right.$ of each orbital *l*, and matrices $M^{\sigma_{l}}$ are obtained from a tensor decomposition of the configuration interaction (CI) coefficient vector.

During the DMRG optimisation procedure, the $M^{\sigma_{l}}$ matrices are optimised one, or, more commonly, two at a time. On each microiteration, truncation based on reduced density matrices ensures that the dimensions of $M^{\sigma_{l}}$ never exceed a pre‐defined parameter *m* named the *maximum bond dimension* or the *number of renormalized block states* and ultimately controls the accuracy of the calculation.

The reduced density matrices, in addition to their essential role for the matrix truncation in DMRG, may be used to calculate the *single‐orbital entropy* and *mutual information*, together referred to as *orbital entanglement measures*.

The single‐orbital von‐Neumann entropy $s_{i}\left(1\right)$ for a given orbital *i* is defined[^70^, ^71^] using the four eigenvalues $\omega_{\alpha ,i}$ of the *one‐orbital reduced density matrix* (1o‐RDM), as

(2) $$s_{i}\left(1\right)=-\sum_{\alpha =1}^{4}\omega_{\alpha ,i}\ln\omega_{\alpha ,i}.$$

The 1o‐RDM for an orbital *i* is contructed by tracing out the states corresponding to orbital occupations of all orbitals except *i*. The single‐orbital entropy measures the entanglement of the orbital *i* with all other orbitals, or in other words, its contribution to the multiconfigurational character of the state. Stein and coworkers[^73^, ^74^, ^75^, ^76^] have devised a method for the active orbital space selection based on single‐orbital entropies. With an appropriate threshold for the single‐orbital entropies, it is possible to perform the selection procedure in a fully‐automated manner. The procedure involves constructing a threshold diagram, i. e. a plot of the number of selected orbitals for a given percentange of the largest single‐orbital entropy (see Figure 2). For each electronic state, the number of orbitals with $s_{i}\left(1\right)$ value larger than a given threshold relative to the maximum value is plotted and an active space is chosen from a plateau on this plot. If the active spaces chosen in this way do not match for each electronic state, the union between all of the state‐specific active spaces is chosen as the final active space.^[76]^

While this approach is effective, it may be quite tedious when many electronic excited states need to be computed. Therefore, here we propose a simplification of this protocol by selecting the *maximum* single‐orbital entropy value for each orbital *i*

(3) $$s_{i}^{\max}\left(1\right)=\max_{k}s_{i}^{k}\left(1\right),$$

where the *k* index runs over all electronic states in question. With maximum orbital entropies at hand we may construct a threshold diagram^[73]^ and select a plateau as in the original approach, resulting in an active orbital space selected for several electronic states.

### Computational Details

**Figure 1:**
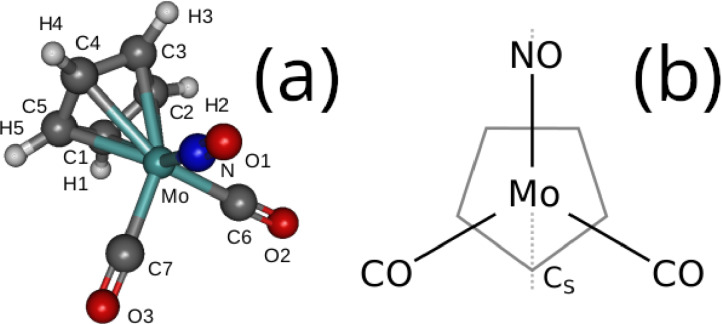
(a) Schematic representation of CpMo(CO)_2_NO with the atom labelling as referred to in Table 1 and (b) top view, showing the staggered configuration of the equilibrium structure.

The equilibrium geometry of CpMo(CO)_2_NO in the electronic ground state was optimized with the B3LYP functional^[84]^ and a def2‐TZVPP basis set.^[85]^ A frequency calculation at the same level of theory confirmed that all the frequencies are positive, and the so‐obtained geometry, with a staggered NO ligand with respect to the Cp‐ring (see Figure 1b), corresponds to a minimum energy structure. The optimization and frequency calculations were performed with the Gaussian16 suite^[86]^ assuming Cs symmetry. For the sake of comparison with the DMRG computations, vertical excitations were also obtained using TD‐DFT at the same level of theory as the geometry optimization. Here the lowest 30 singlet excited states were computed. The TD‐DFT calculations were performed with the ORCA 4.2.1[^87^, ^88^] program package, employing the RIJCOSX^[89]^ approximation.

The initial active space in the DMRG calculation was built from the molecular orbitals calculated with the Hartree‐Fock (HF) method and the ANO‐RCC‐MB basis set.^[90]^ The electronic ground state of CpMo(CO)_2_NO has 120 electrons occupying 60 orbitals in a HF single determinant picture. Using the HF orbital basis, a DMRG‐CI calculation was performed with the 20 lowest‐lying core orbitals as frozen, 21 orbitals (comprising 3d, 4s, 4p orbitals of Mo and lowest‐lying valence *σ* ligand orbitals) as inactive and 30 orbitals, comprising *σ*‐, *π*‐, and *δ*‐type orbitals with interactions between the ligands and Mo, as well as orbitals of the *π*‐ and *σ*‐ interactions inherent to the ligands, accommodating 38 electrons in the active space (thereby referred as (30,38) active space, see below). Single‐orbital entropies have been calculated[^71^, ^72^] and analysed with the autoCAS[^73^, ^74^, ^75^, ^76^] program to reduce the active space to 30 electrons in 26 orbitals (30,26) using a procedure explained in the following section. DMRG‐SCF calculations were performed subsequently with the reduced active space, employing the ANO‐RCC‐VTZP^[90]^ basis set. The DMRG calculations will be referred using the general notation DMRG‐CI(*n*_electrons_,*n*_orbitals_)[*m*] or DMRG‐SCF(*n*_electrons_,*n*_orbitals_)[*m*], respectively, where we have used maximum bond dimensions (*m*) of 250 and 1000. TD‐DFT calculations show that the key states involved in the photodissociation of both CO and NO upon irradiation with visible and low‐energy UV light are the lower‐lying excited states. For this reason, and to spare computational effort, only seven lowest‐lying excited singlet states were considered within the DMRG calculations. All DMRG calculations have been done using the QC‐MAQUIS DMRG program^[91]^ version 2.1 and 3.0, integrated into the OpenMolcas program package.^[92]^

The character of the electronic excited states was analysed with natural transition orbitals (NTOs)^[93]^ and in terms of charge‐transfer numbers using the TheoDORE analysis suite.^[94]^ The latter procedure enables automatic quantitative wavefunction analysis in terms of the amount of charge transfer associated to the electron excited, using predefined molecular fragments.^[95]^

## 2 Results and Discussion

### 2.1 Molecular Structure

Relevant geometrical parameters of the complex are compiled in Table 1 and compared to the experimental data.^[96]^ Most calculated values agree within 2 %, except for the Mo−CO and Mo−NO bond ligands, which are within 5 % of the experimental values. Small deviations away from the Cs symmetry can be attributed to the crystalline nature of the complex in the experiment, while our calculations are done in gas phase.

**Table 1:** Calculated (calc) bond lengths [Å] and angles [°] and experimental values (exp) (taken from Ref. [96])

bond or angle	calc	exp
Mo−N	1.817	1.899
Mo−C7	1.998	1.957
Mo−C8	1.998	1.941
<Mo−N−O1	180.	177.85
N−O1	1.170	1.167
<Mo−C6‐O2	180.	178.21
<Mo−C7‐O3	180.	176.80
C6‐O2	1.151	1.143
C7‐O3	1.151	1.154

The NO ligand is coordinating to Mo in a linear fashion, which is often the case for closed‐shell singlet nitrosyl complexes and is therefore typically formally considered as a NO^+^ cation. This is further supported by the fact that the CO ligands are also linearly coordinated and NO^+^ is isoelectronic to CO. To obtain further information about the NO coordination from the multiconfigurational wavefunctions, we have analysed the natural orbital occupation numbers in the DMRG‐SCF(30,26)[1000] calculation (see below). The total occupation number of the frontier NO ligand orbitals, as well as the total occupation number of the Mo *d* orbitals both amount to 6.04, which is consistent with the description of the Mo centre as *d* ^6^ and NO as NO^+^.

### 2.2 Selection and Reduction of the DMRG Active Orbital Space

**Figure 2:**
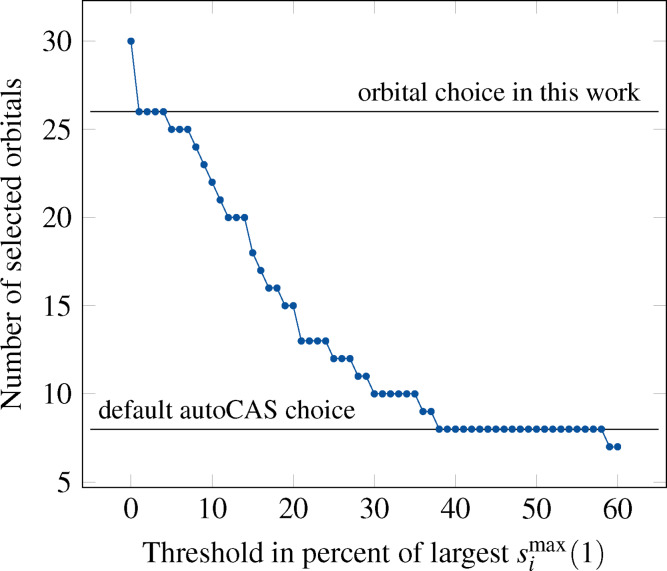
Threshold diagram constructed with the maximum single‐orbital entropy values.

Although the initial active space of 38 electrons in 30 orbitals is computationally feasible for DMRG‐SCF, it makes sense to reduce this active space by eliminating orbitals with small contributions to the static correlation. To this aim, we employed a threshold diagram, i. e. a plot of the number of selected orbitals for a given percentange of the largest maximum single‐orbital entropy, see Figure 2.

In this work, we chose a very conservative threshold of 2 %, which amounts to the first plateau in Figure 2, as we wish also to account for the effect of dynamic correlation in our DMRG calculations. We are still consistent with the statement in Ref. [73], which says that orbitals may be safely excluded from the active space if their single‐orbital entropy is below 2 % of that of the orbital with the highest single‐orbital entropy. As can be seen from Figure 2, this results in excluding four orbitals (51, 52, 54, 55 in Figure S1 of the Supporting information), all of which represent *σ*‐type orbitals centered on the cyclopentadienyl ring, and are situated well below the HOMO in the HF orbital picture. After reduction, the active space size amounts to 26 orbitals, which allocate 30 electrons.

We note that this selected active space is the same that if one would use the original autoCAS approach for excited states^[76]^ instead of our selection procedure, provided the $s_{i}\left(1\right)$ selection threshold is the same. This shows that our approach is robust and reliable, while it has the advantage that a threshold diagram must be constructed only once, and the single‐orbital entropy of each state are considered on equal footing.

### 2.3 Selection of the Basis Set and DMRG *m*‐Parameter

Besides the careful choice of the active space, DMRG‐SCF calculations also require a sensible basis set and a maximum bond dimension (*m*) in order to achieve a reasonable balance between accuracy and computational cost. The *m* value need not to be held constant during the course of the DMRG‐SCF optimization, and a DMRG‐CI calculation employing orbitals from an earlier DMRG‐SCF calculation with a smaller *m* value may significantly reduce computational cost with only a small penalty for accuracy. To assess the effect of the active space, basis set and the *m* value, we recorded the excitation energies for DMRG calculations before and after the active space reduction and with different basis sets and *m* values in Table 2. The reduction of the active space (the difference between columns (a) and (b)) does not manifest a large excitation energy shift, which is at most 0.2 eV. A much larger effect is seen in expanding the basis set from the minimal basis to the triple‐zeta basis (column (c)), where the excitation energy is shifted by up to 0.47 eV compared to the same calculation but with the minimal basis (column (b)). The increase of *m* from 250 to 1000 (column (d)) causes a significant red‐shift of S_4_, S_6_ and S_7_ by 0.22 eV on average, but a much smaller effect on other excited states. Finally, performing the complete DMRG‐SCF optimisation with *m*=1000 (column(e)) shifts the excitation energies only by a small amount, namely below 0.1 eV for all excited states: this indicates that a large value for *m* is important for quantitative estimation of excitation energies, however intermediate iterations in DMRG‐SCF may be carried out with a smaller value.

**Table 2:** Excitation energies for various DMRG‐based methods: (a): DMRG‐CI(38,30)[250]/MB (b): DMRG‐SCF(30,26)[250]/MB (c): DMRG‐SCF(30,26)[250]/TZ (d): DMRG‐CI(30,26)[1000], employing DMRG‐SCF(30,26)[250]/TZ orbitals (e): DMRG‐SCF(30,26)[1000]/TZ. MB and TZ denote ANO‐RCC‐MB and ANO‐RCC‐VTZP basis sets, respectively.

State	a	b	c	d	e
S_1_	3.09	3.22	3.40	3.30	3.32
S_2_	3.36	3.27	3.74	3.65	3.66
S_3_	3.36	3.45	3.83	3.83	3.78
S_4_	3.97	4.03	4.31	4.11	4.13
S_5_	3.99	4.18	4.41	4.38	4.36
S_6_	5.07	5.22	4.97	4.76	4.81
S_7_	5.33	5.53	5.67	5.43	5.45

### 2.4 Analysis of the Low‐Lying Excited States of CpMo(CO)_2_NO

Here we shall analyse the characters of the seven lowest excited states obtained with DMRG‐SCF(30,26)[1000] and TD‐DFT, in terms of the NTOs,^[93]^ see Table 3. DMRG‐SCF yields excitations from Mo *d* orbitals, often with contributions of the bonding interaction with $\pi {}^{*}$ orbitals of CO or NO, to an anti‐bonding linear combination of Mo *d* orbitals and either a $\pi_{\mathrm{NO}}{}^{*}$ or $\sigma_{\mathrm{CO}}{}^{*}$ orbitals. The lowest five excited states show an excitation into Mo−NO antibonding orbitals with a $\pi {}^{*}$ character, and S_6_ and S_7_ are excitations into Mo−CO σ* antibonding orbitals. The high‐density of low‐lying dissociative excited states indicates that CpMo(CO)${}_{2}$ NO could be a promising photoCORM and a photoNORM compound simultaneously. The oscillator strengths (Table 4) reveal that at least one of the NO‐antibonding states (S_4_) and both CO‐antibonding S_6_ and S_7_ are bright, allowing access to potential dissociative states after excitation.

**Table 3:**
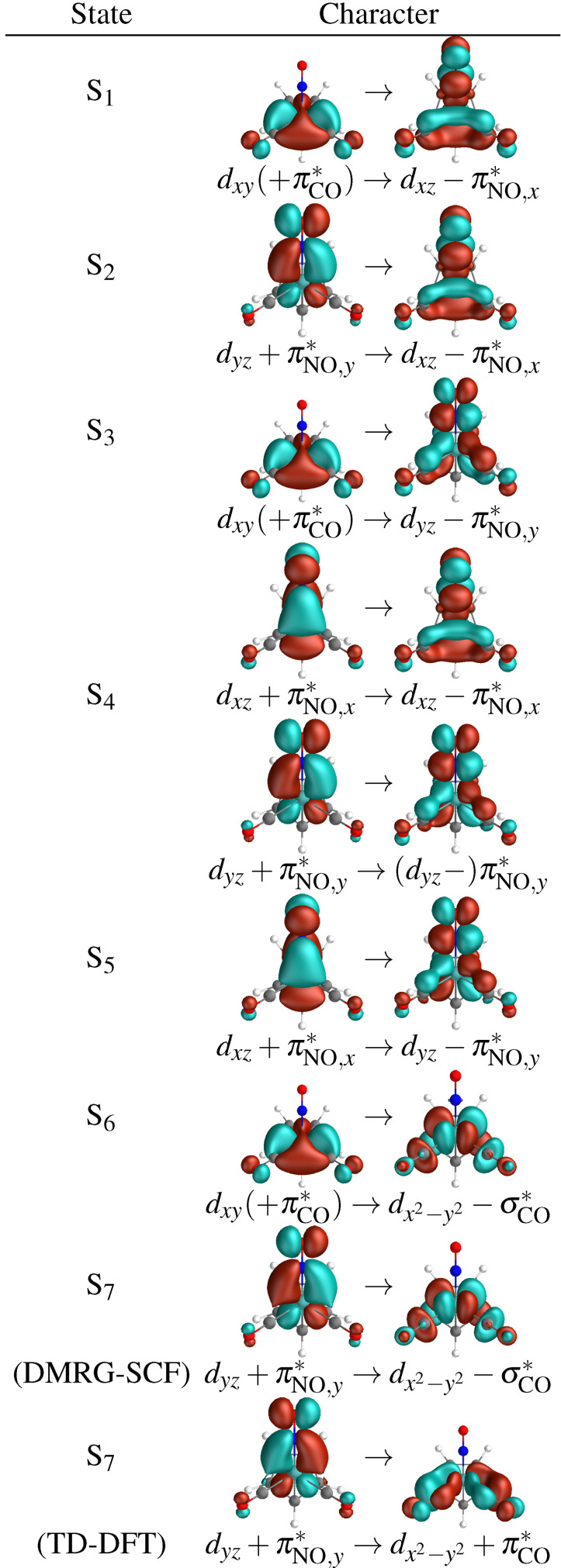
Excited state characters from natural transition orbitals for the first seven excited singlet states obtained with TD‐DFT and DMRG‐SCF(30,26)[1000]. Minor contributions are shown in brackets. S_4_ consists of almost equal NTO contributions of each of the lines. The only major difference between TD‐DFT and DMRG‐SCF character is S_7_.

**Table 4:** Excitation energies in eV and oscillator strengths for the first seven excited singlet states obtained with DMRG‐SCF(30,26)[1000] and TD‐DFT.

State	DMRG‐SCF(30,26)[1000]		TD‐DFT	
	Δ*E*	*f*	Δ*E*/eV	*f*
S_1_	3.32	0.0011	2.90	0.0011
S_2_	3.66	0.0057	2.96	0.0010
S_3_	3.78	0.0005	3.27	0.0
S_4_	4.13	0.0117	3.43	0.0042
S_5_	4.36	0.0080	3.70	0.0008
S_6_	4.81	0.0188	4.20	0.0125
S_7_	5.45	0.0141	4.49	0.0147

The energy grouping of the states with antibonding character (NO−S_1_ to S_5_, CO−S_6_ and S_7_, with a gap of almost 0.5 eV between S_5_ and S_6_) are promising for the ability to release selectively CO or NO in CpMo(CO)_2_NO by excitation with different wavelengths. However, as the dissociation mechanisms heavily depend on energetic barriers and the capability for internal conversion to the lower‐lying excited states, further studies on the processes occurring directly after photoexcitation must be conducted to determine the feasibility of the dissociation control.

**Figure 3:**
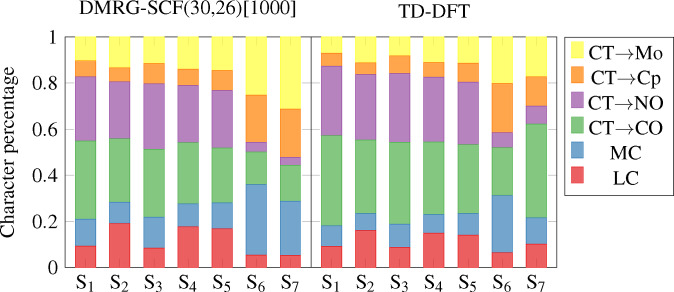
Decomposition of charge‐transfer numbers into various classes of excitations for DMRG‐SCF(30,26)[1000] and TD‐DFT.

Additional insight into the character of the electronic excited states can be obtained from the decomposition of the charge‐transfer numbers,^[94]^ which allow for quantification of charge transfer between pre‐defined molecular fragments. In this analysis, the complex has been partitioned into the Mo center and the four Cp, NO and CO ligands. With this definition, we may quantitatively determine the percentage of charge‐transfer and local character for each excitation. The decomposition of charge‐transfer numbers for a set of fragments for each ligand and the metal is shown in Figure 3, where local excitation characters within the same fragment are labelled as ligand‐ and metal‐centered (LC and MC), respectively, and charge‐transfer characters to various ligands and the metal are labelled as CT.

The differences between state characters of S_1_–S_5_ and S_6_–S_7_, found in the NTO characters, is confirmed in distinct patterns for charge‐transfer numbers. S_1_ to S_5_, identified as potentially NO‐dissociative from the NTO characters, show a significant contribution (up to 28 % in DMRG‐SCF, up to 30 % in TD‐DFT) of charge transfer towards the NO ligand. Charge transfer towards the CO ligand is, however, also significantly present in these states (up to 39 % cumulatively for the two CO ligands). S_6_ and S_7_, identified as CO‐dissociative by the NTO characters, show a significant metal‐centered excitation character. The charge‐transfer to NO drops significantly to below 5 % (for DMRG‐SCF) in these states. The charge‐transfer to CO drops as well, but to a much lesser extent, while at the same time charge transfer to the metal and the Cp ligand increase.

The largest difference between TD‐DFT and DMRG‐SCF characters is found in S_7_: while DMRG‐SCF predicts it with a similar metal‐centered character to S_6_, in TD‐DFT it has a significantly larger charge‐transfer contribution to the CO ligands and less charge transfer to the metal. The discrepancy can also be seen in the NTO characters, containing a contribution of an excitation to a Mo−CO σ* antibonding orbital in DMRG‐SCF, but consisting mainly an d→d excitation into a $d_{x^{2}-y^{2}}$ orbital of Mo in TD‐DFT. Furthermore, the S_7_ state shows also by far the largest discrepancy of excitation energy between DMRG‐SCF and TD‐DFT, namely almost 1 eV. One reason for this discrepancy might be a possible large double‐excitation character of this state: a proof of this hypothesis requires the analysis of double excitations with DMRG‐SCF, which is beyond the scope of this work.

Other excited states show surprisingly similar characters for both DMRG‐SCF and TD‐DFT, both in NTO characters and charge‐transfer numbers. The excitation energies in TD‐DFT are red‐shifted by 0.4–0.7 eV with respect to the DMRG‐SCF energies (see Table 4).

## 3 Conclusions

In this work we computed the excitation energies, oscillator strengths and state characters of the low‐lying excited states of CpMo(CO)_2_NO with DMRG‐SCF. For the selection of the active space in the DMRG‐SCF calculation for multiple excited states, we used an algorithm that employs the maximum single‐orbital entropy of all states. This approach is robust with respect to the original active space selection protocol by Stein et al.^[76]^ but is simpler to implement and considers several electronic excited states on one go.

From the so‐reduced active space‐DMRG(30,26) calculations, we found that the five lowest‐lying states of CpMo(CO)_2_NO contain significant contributions of excitations to a metal‐NO antibonding orbital and charge transfer to the NO ligand, offering opportunities for the dissociation of the NO ligand. One of these states shows an absorption peak in the violet range, thus allowing for exciting directly an NO‐antibonding orbital with visible light. Higher‐lying excited states are metal‐centered excitations with contributions to metal‐CO antibonding orbitals, facilitating the CO dissociation upon photoexcitation at lower wavelengths. Comparing with TD‐DFT, DMRG‐SCF excitation energies are red‐shifted by 0.4–1.0 eV, and the state characters agree very well between both methods.

The present DMRG‐SCF calculation may serve as a reference for benchmarking multiconfigurational calculations with smaller active spaces required for further studies of the photodissociation mechanism of CpMo(CO)_2_NO and other photoCORMs and photoNORMs.

## Conflict of interest

The authors declare no conflict of interest.

## Supporting information

As a service to our authors and readers, this journal provides supporting information supplied by the authors. Such materials are peer reviewed and may be re‐organized for online delivery, but are not copy‐edited or typeset. Technical support issues arising from supporting information (other than missing files) should be addressed to the authors.

Supporting Information
