# Supplementary material for: A Density Matrix Renormalization Group Study of the Low‐Lying Excited States of a Molybdenum Carbonyl‐Nitrosyl Complex
Source: Chemphyschem. 2021 Oct 12;22(22):2371–7. doi: 10.1002/cphc.202100549 (PMC9292996; doi:10.1002/cphc.202100549)
Supplement: Supplementary file 1 — Supporting Information [file CPHC-22-2371-s001.pdf]

ChemPhysChem

Supporting Information

A Density Matrix Renormalization Group Study of the Low-Lying Excited States of a Molybdenum Carbonyl-Nitrosyl Complex

Leon Freitag, Leopold Lindenbauer, Markus Oppel, and Leticia González*

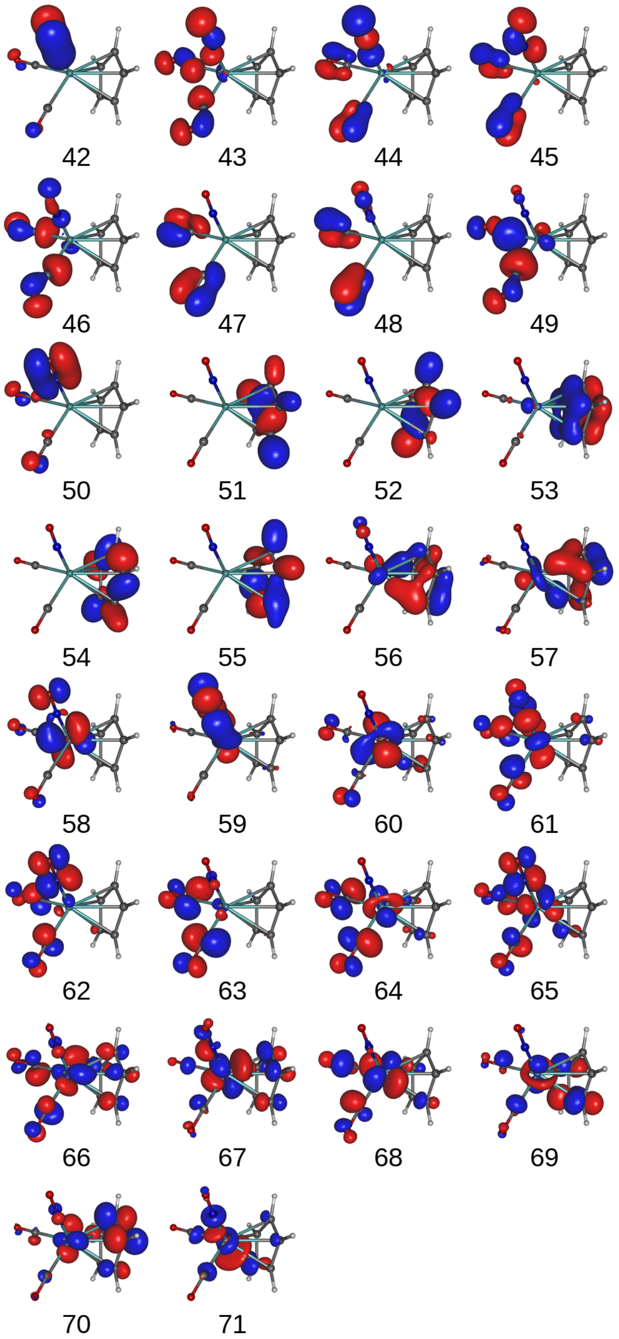

Figure S1: Molecular orbitals used in the initial (38, 30) active space of the DMRG-CI calculation. Orbital 60 is the highest occupied molecular orbital in the electronic ground state.

Table S1: Maximum state-specific single-orbital entropies obtained from the initial DMRG(38,30)[250]-CI calculation and the DMRG(30,26)[250]-CI calculation. Values below the 2% threshold are highlighted in bold.

Orbital Number	DMRG(38,30) $s_i^{max}(1)$	DMRG(30,26) $s_i^{max}(1)$
42	0.2665	0.2490
43	0.1008	0.1139
44	0.1390	0.1398
45	0.1825	0.1749
46	0.1060	0.1092
47	0.1904	0.1631
48	0.2160	0.1807
49	0.1244	0.1248
50	0.2641	0.2530
51	**0.0089**	-
52	**0.0073**	-
53	0.1436	0.0923
54	**0.0034**	-
55	**0.0034**	-
56	0.3845	0.3425
57	0.4511	0.3771
58	1.1829	1.1311
59	1.1408	1.1458
60	1.0482	0.9850
61	1.2830	1.2035
62	1.1783	1.1751
63	1.1437	1.1324
64	0.3559	0.2501
65	0.7550	0.8217
66	1.0669	1.0681
67	0.2359	0.2207
68	0.4868	0.6984
69	0.1951	0.1482
70	0.3203	0.3389
71	0.0605	0.0920

Table S2: Single-orbital entropies for all 8 roots obtained from the initial DMRG-CI(38,30)[250] calculation

Orbital no.	S_0	S_1	S_2	S_3	S_4	S_5	S_6	S_7
42	0.1404	0.1652	0.1192	0.2665	0.1778	0.1518	0.1365	0.2149
43	0.0876	0.0978	0.1008	0.0848	0.0878	0.0908	0.0905	0.0739
44	0.1153	0.1390	0.1390	0.1142	0.1079	0.1083	0.1320	0.1004
45	0.1648	0.1299	0.1461	0.1486	0.1723	0.1825	0.1562	0.1689
46	0.0988	0.1026	0.1060	0.0962	0.0955	0.0989	0.0963	0.0938
47	0.1588	0.1904	0.1900	0.1633	0.1549	0.1567	0.1710	0.1300
48	0.1889	0.1655	0.1564	0.2023	0.1803	0.1776	0.1807	0.2160
49	0.1044	0.1107	0.1109	0.1231	0.1101	0.1100	0.0910	0.1244
50	0.1798	0.1332	0.1902	0.1559	0.2367	0.2641	0.1800	0.1688
51	0.0072	0.0070	0.0070	0.0082	0.0072	0.0072	0.0067	0.0089
52	0.0061	0.0072	0.0073	0.0060	0.0061	0.0065	0.0069	0.0057
53	0.1408	0.1365	0.1415	0.1401	0.1342	0.1408	0.1436	0.1375
54	0.0031	0.0031	0.0034	0.0030	0.0029	0.0032	0.0031	0.0027
55	0.0030	0.0027	0.0027	0.0034	0.0027	0.0028	0.0028	0.0031
56	0.2563	0.2802	0.2607	0.3845	0.2764	0.2502	0.2779	0.3609
57	0.2220	0.4511	0.4431	0.2260	0.2251	0.2335	0.4112	0.2302
58	0.5580	0.3794	0.3620	0.3269	1.1829	1.1129	0.5524	0.8174
59	0.5710	0.8151	0.6748	1.1408	0.9945	0.6987	0.5973	1.0744
60	0.2807	1.0318	1.0248	0.2987	0.3275	0.3595	1.0482	0.2974
61	0.5891	0.8195	1.1624	0.4612	1.2467	1.2830	0.6380	0.4857
62	0.4868	1.0574	0.3682	1.1783	1.0042	0.6677	0.4572	0.7217
63	0.2641	0.2585	0.2813	0.2991	0.3174	0.3565	1.0371	1.1437
64	0.2954	0.2483	0.2490	0.2848	0.2947	0.3042	0.2775	0.3559
65	0.4066	0.5823	0.3473	0.7550	0.4896	0.3637	0.4162	0.4540
66	0.2686	0.3068	0.3296	0.2595	0.3053	0.3427	1.0669	0.4634
67	0.1868	0.2215	0.1756	0.2359	0.2067	0.1927	0.1860	0.2206
68	0.2067	0.2306	0.2500	0.1955	0.2399	0.2644	0.4868	0.4130
69	0.1884	0.1828	0.1831	0.1951	0.1798	0.1832	0.1916	0.1823
70	0.2126	0.2335	0.2599	0.1980	0.2277	0.2491	0.3203	0.2117
71	0.0588	0.0582	0.0552	0.0605	0.0541	0.0554	0.0532	0.0573
